# In Situ Passive Sampling to Monitor Long Term Cap Effectiveness at a Tidally Influenced Shoreline

**DOI:** 10.3390/toxics10030106

**Published:** 2022-02-23

**Authors:** Alex V. Smith, Xiaolong Shen, Uriel Garza-Rubalcava, William Gardiner, Danny Reible

**Affiliations:** 1 Department of Civil, Environmental and Construction Engineering, Texas Tech University, Lubbock, TX 79409, USA; alex.v.smith@ttu.edu; 2 Department of Chemical Engineering, Texas Tech University, Lubbock, TX 79409, USA; xiaolong.shen@ttu.edu (X.S.); u.garza-rubalcava@ttu.edu (U.G.-R.); 3 U.S. Army Corps of Engineers, Seattle District Seattle, Washington, DC 98134, USA; william.w.gardiner@usace.army.mil

**Keywords:** passive sampling, SPME, polycyclic aromatic hydrocarbons

## Abstract

Polydimethylsiloxane solid-phase microextraction passive samplers were used to evaluate long-term performance of a sand/gravel cap placed in 2005 in a tidally influenced shoreline in Puget Sound to reduce polycyclic aromatic hydrocarbon (PAH) transport into overlying surface water. Sampling in both 2010 and 2018 measured porewater concentrations of <1 ng/L total PAHs in the cap layer. d-PAH performance reference compounds were used to evaluate the extent of equilibration of the contaminants onto the samplers and to estimate net upwelling velocities through a mass-transfer model. The upwelling velocities were used to predict long-term migration of selected PAHs through the cap, showing that the cap is expected to continue being effective at limiting exposure of contaminants at the cap–water interface.

## 1. Introduction

Contaminated sediment caps physically isolate environmental contamination and delay or eliminate significant release of hydrophobic organic compounds (HOCs). In the absence of advection, transport below surface layers is driven by sorption-retarded diffusion and long-term effectiveness is typically observed [1,2]. Sediment caps are challenged, however, in environments where advective transport such as groundwater upwelling or tidal pumping of groundwater is important. Evaluating the effect of these processes is a critical component of long-term monitoring of cap effectiveness.

Freely dissolved concentrations of HOCs can be measured via passive sampling, e.g., by solid-phase microextraction (SPME) utilizing polydimethylsiloxane (PDMS) as a sorbent [3]. Freely dissolved concentrations provide an indication of the contaminant that is mobile and available in a stable sediment cap. Previous studies have demonstrated the use of passive sampling to estimate concentrations influencing the benthic community in surficial sediments [4,5], and calculating the diffusive flux at the sediment–water interface [6,7].

A 1–2 m sand and gravel cap was placed in 2005 in the area offshore of a former creosote-processing facility (Puget Sound Resources) to contain polycyclic aromatic hydrocarbons (PAHs). The site is subject to both groundwater upwelling and tidal variations that would be expected to lead to tidal pumping of groundwater. Passive-sampling measurements in 2010 [8] showed that 5 years after placement, porewater concentrations over the upper 90 cm of the cap layer were not significantly different from concentrations in the overlying water. In a few locations, near-surface concentrations (<10 cm) were slightly above surface-water concentrations, either due to the deposition of sediments containing PAHs or intermixing of contaminated sediments during placement. There was no evidence at that time of significant PAH contamination relative to concentration levels of concern, or evidence of migration of PAHs through the cap.

In this study, PDMS SPME fibers were used to evaluate the long-term performance of a sediment cap by measuring porewater concentration profiles in 2018, 13 years after cap placement. Performance reference compounds were used to estimate both the extent of equilibration and upwelling velocities through application of a porous media mass-transfer model based on Kimura’s theory [9]. The upwelling velocities and measured site porewater/sediment partitioning were used to predict long-term migration of PAHs in the sediment cap using CapSim [10], a model of near-surface contaminant transport in sediments. The study provides evidence of the long-term performance of a sand and gravel sediment cap in an environment subject to groundwater upwelling and tidal pumping, and demonstrates the use of performance reference compounds to estimate both the equilibrium for passive-sampling evaluation of cap performance and the magnitude of groundwater upwelling.

## 2. Materials and Methods

### 2.1. PDMS SPME Fibers, Sampling Devices, and PRCs

The PDMS SPME fibers were fabricated by Polymicro Technologies (Phoenix, AZ, USA). The fibers were composed of a 562 µm outer diameter of PDMS coating on a 485 µm glass core for a volume of 0.64 µL/cm of PDMS. Prior to deployment, the PDMS fibers were cut into 90 cm segments, washed twice with hexane, acetonitrile, and methylene chloride sequentially for 30 min each, rinsed with ultrapure distilled deionized water (DDI), and dried with lint-free tissue. Only high-purity solvents were used in the preparation process of these PDMS fibers. 

This study utilized the use of PRCs to evaluate the equilibration of the target compounds, as well as estimate effective mass-transfer coefficient in the media surrounding the sampler for purposes of estimating groundwater upwelling velocities. Deuterated PAHs, d10-fluoranthene, d12-chrysene, d12-benzo[b]fluoranthene, and d14-dibenzo[a,h]anthracene, spanning the range of hydrophobicity of the target compounds were used as PRCs. The deuterated PAHs were purchased from Cambridge Isotope Labs (Tewksbury, MA, USA). To load the PRCs on the fibers, cleaned fibers were inserted into a methanol/water mix (20:80 *v*/*v*) that was spiked with the four d-PAH PRCs and left to equilibrate for a minimum of 28 days on a shaker table.

For ease of insertion and protection from sand and gravel in the sediments, the fibers were placed in stainless-steel Henry PushPoint samplers (M.H.E. Products, East Tawas, MI, USA) with a ¼” (6.5 mm) outer tube and smaller-diameter inner rod. The sampler was modified by perforating the outer (shield) tube along its length with 4 mm-diameter holes and cutting a slit along the length of the inner rod to which the fibers were secured by using waterproof silicone (caulk) at the two ends of the fiber. Prior to use, the stainless-steel samplers were washed with hot water and detergent, soaked sequentially in hexane, acetonitrile, and methylene chloride (Thermo Fisher Scientific, Waltham, MA, USA), flushed with deionized water, and dried at 105 °C overnight.

The approaches to passive sampling in 2010 and 2018 were essentially identical, except that both thicker and thinner PDMS fibers were employed in 2010 to allow estimation of equilibration, while PRCs were employed for the same purpose in 2018 [4,8].

### 2.2. Site and Sampling Design

**Figure 1:**
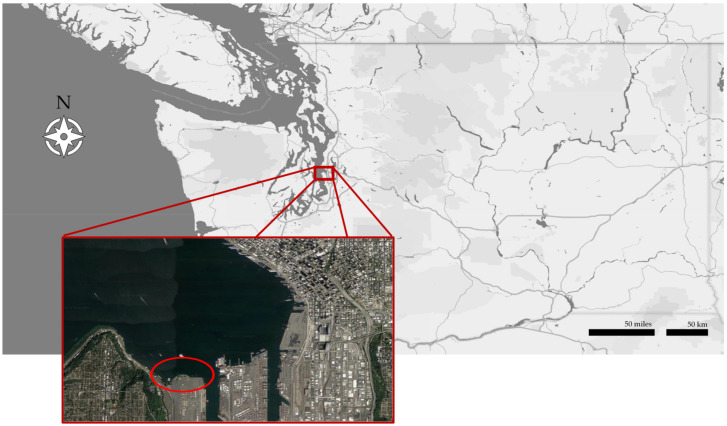
Location of Puget Sound Resources in West Seattle, Washington. The ellipse shows the location of the site. Imagery ©2022 CNES/Airbus, Landsat/Copernicus, Maxar Technologies, U.S. Geological Survey, USDA Farm Service Agency, Map data ©2022.

The subtidal sediments at the former Pacific Sound Resources site in West Seattle along the southern shore of Puget Sound were capped with sand- and gravel-borrow materials in 2005 (Figure 1). These sediments had been impacted by creosote-containing PAHs migrating through the subsurface. A slurry wall was also placed at the shoreline to control any further migration from upland areas.

**Figure 2:**
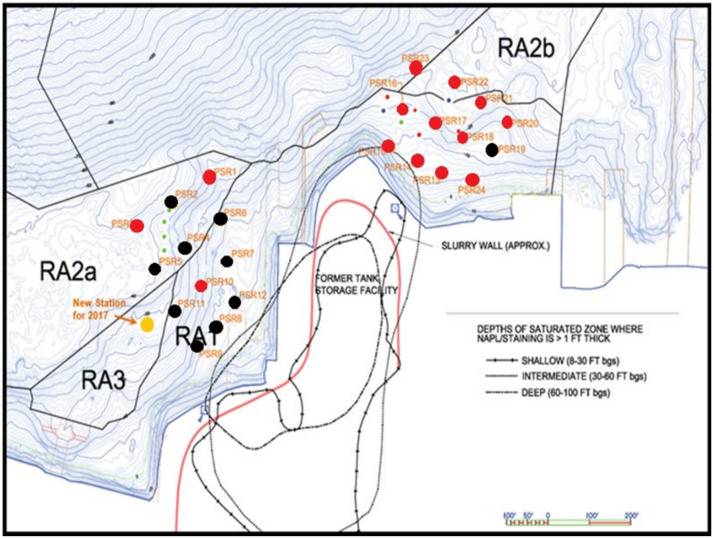
SPME sampling locations for the 2010 and 2018 sampling event at Puget Sound Resources Superfund Site. SPME sampling locations that had an increase in porewater concentration in 2018 compared to 2010 are marked red; those that did not increase are black. Penetrations refer to depth probing of the cap several years after its placement.

**Figure 3:**
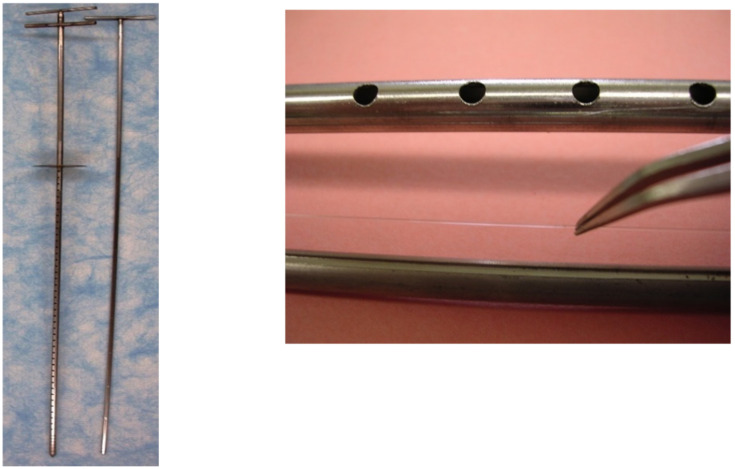
SPME sampler with a 90 cm working section showing perforated outer shield tube and inner rod (**left**). Close-up of the perforated shield tube; the inner rod with slit and the PDMS are also shown (**right**). The fiber has 0.59 µL/cm of PDMS around a 497 µm-diameter glass core.

The cap material contained approximately 0.3% organic carbon and was placed to a thickness from 1 m (offshore) to 2 m (near-shore). The site is subject to a typical tidal range of 2–3 m. In situ SPME passive samplers were placed at 24 individual monitoring locations along seven transects extending out from the coastline in 2010 and again in 2018. Sample locations 1–12 are in the northwestern part of the site (NW) along three transects, while 13–24 are in the northeastern portion (NE) along an additional three transects, as shown in Figure 2. Samplers were deployed at these locations on 13–14 March 2018, and 22 of the 24 locations were retrieved on 28–29 March 2018. A sampler could not be deployed at location 9, and the sampler at location 8 was found on the sediment surface upon retrieval. A sampler was also placed at a new location in 2018. Locations 8, 9, and the new location were all located on the western edge of the sampling array. No data could be retrieved from samplers at locations 8 and 9, but a nearby location showed low PAH concentrations in porewaters (∑PAH = 138 ng/L) consistent with other locations in the NE sampling area. Three samplers were suspended in the surface water: two in the water above the site and one in a reference location well away from the site. Figure 3 shows the SPME sampler used at the site.

As noted previously, the measurements collected in 2010 showed no evidence of migration of PAHs within the upper 90 cm of the cap layer, and porewater concentrations measured at that time were essentially uniform and approximately equal to that measured in the overlying water, except for slightly higher (2–3 times surface water concentrations) in the near surface (upper 10 cm) of 10–20% of the sampling locations. Because of the lack of significant contamination observed during the 2010 sampling, the analysis herein will focus on the 2018 sampling program, although the results will be compared to the 2010 data.

### 2.3. Chemical Analysis

Upon retrieval, the stainless-steel PushPoint samplers were dismantled and the PDMS fibers were removed from the stainless-steel housing and wiped clean with DDI water to remove any residual sediment from the fiber. Once all visible organic matter was removed, the SPME fibers were sectioned into adjacent 2 cm segments for every target depth between the 90 cm exposure section. All fibers were placed in autosampler vials prefilled with 200 µL of acetonitrile immediately after segmentation from the main fiber length and shipped back to Texas Tech University (Lubbock, TX, USA) for processing. Previous studies had shown that extraction of contaminants from the fibers is essentially complete within 24 h.

Once received, the PDMS-solvent extracts were analyzed using high-performance liquid chromatography (HPLC, Agilent 1260, Agilent Technologies, Santa Clara, CA, USA) in accordance with EPA method 8310, SW-846 3rd edition [11], with fluorescence detection (FLD). The target compounds included the deuterated PAHs serving as PRCs, the 16 priority pollutant PAHs [12], 2 methylnaphthalene (2-MNP), and dibenzofuran (DBF). Of the 16 priority PAHs, acenaphthylene is not detectable by FLD and benzo[g,h,i]perylene and indeno[1,2,3-cd]pyrene coelute and were reported as a sum of both compounds. The sum of the 13 measured PAHs, the combined coeluting compounds, plus 2-MNP and DBF are referred to here as ∑PAH. Chromatographic separation was conducted using a 1.0 mL/min isocratic flow composed of 3:7 (*v*/*v*) water:acetonitrile at 40 °C, and retention times for the target compounds are shown in the Supplementary Materials.

Calibration standards were prepared from stock solutions (Cambridge Isotope Labs) ranging from 0.5 µg/L to 100 µg/L. Calibrations were linear with r^2^ > 0.999 and a relative standard deviation (RSD) of <20% at all concentrations. For every 10 field samples analyzed, a 5 µg/L or 10 µg/L calibration quality check (QC) standard confirmed stability of the calibration. Equipment blanks (EBs) containing only acetonitrile were run periodically throughout the sequence to ensure no background interferences were present.

### 2.4. Determination of the Freely Dissolved Concentration

The estimation of the freely dissolved porewater concentration ($C_{pw}$, [ng/L]) using SPME PDMS passive samplers involves the quantification of the concentration sorbed to the polymer ($C_{PDMS}$ [ng/cm], Equation (1)) and the unitless polymer–water partitioning coefficient ($K_{pw}$, Equation (2)) that assumes linear and reversible uptake to the target compounds and correlates with hydrophobicity, as indicated by the octanol–water partitioning coefficient ($K_{ow}$) [13]. The mass on the polymer is calculated based on the specific geometry of the sampler and amount of solvent used to extract the contaminates of concern.

(1) $$C_{PDMS}=\frac{A*RSF_{HOC}*V_{s}}{L_{f}*V_{f}}$$

where A represents the integration area from a specific compound from the HPLC, $RSF_{HOC}$ is the response factor from a standard calibration curve unique to each chemical class of HOCs, $V_{s}$ is the volume of solvent used [mL], $V_{f}$ is the volume of PDMS on the fiber per unit length (0.585 µL/cm), and $L_{f}$ is the length of fiber [cm]. The freely dissolved porewater concentration, $C_{pw}$, is given by Equation (2).

(2) $$C_{pw}=\frac{C_{PDMS}}{K_{pw}*f_{ss}}$$

where $K_{pw}$ represents the polymer–water partitioning coefficient for PAHs (Ghosh et al. [14]). The $f_{ss}$ calculation uses the observed release of PRCs and assumes reversible desorption, which effectively recognizes that half of the PRC initially loaded onto the fiber would be released during the time that an equivalent target compound has achieved 50% equilibration during uptake. The extrapolation to other PAH compounds follows the methodology described in Shen and Reible [15] that takes into account the cylindrical geometry of the fiber.

Both PRC release and target-compound uptake are expected to be controlled by external mass-transfer processes and sediment–water partitioning [16], and thus the PRC release data could also be used to estimate mass transfer in the sediment surrounding the sampler (discussed below).

### 2.5. CapSim Modeling

A software-modeling tool, CapSim [10] was used to predict contamination migration in the future based on the measured concentrations and estimated upwelling velocities. The model accounts for multiple layers of varying properties and allows the user to input specific properties of the material or use typical values from a database of characteristics for different sediment and capping materials. The model incorporates traditional porous-media transport processes including advection, diffusion, dispersion, reaction, and sorption, but also includes the capability to simulate processes specific to the near-surface sediment including deposition, consolidation, bioturbation, and exchange with the overlying water [10]. For the purposes of the current simulation, the measured porewater concentrations in 2018 were used as initial conditions. Default parameters were used in the model, except for two site-specific parameters: groundwater upwelling rates, and partition coefficients in the cap materials and near-surface sediments. Groundwater upwelling rates were estimated from the PRC data (described below) while paired sediment and porewater samples were collected in the near surface (0–10 cm) of a nearby Puget Sound site that was also capped with sand and gravel at approximately the same time as this site. This data was used to estimate partitioning and organic carbon content in near-surface sediments [17]. Partitioning in the lower portion of the cap (>10 cm depth) was estimated from contaminant organic carbon-based partition coefficients and the original organic carbon content (0.3%) of the placed cap material. Changes were not expected to sorption or organic carbon content in the deeper parts of the cap. Only the surface layer of the cap would be affected by sediment deposition and near-surface mixing processes such as bioturbation. Other model parameters which would have a minor impact on contaminant migration over most of the cap either employed model defaults or were directly estimable from the site characteristics [2]. CapSim, as well as input and output files for these simulations, are available from the corresponding author.

## 3. Results

### 3.1. Remedy Effectiveness

As stated previously, the sampling area was split into the NE and NW shoreline with the tip of the isthmus being the divider. Samplers 1–12 were within the NW sampling region while samplers 13–24 were located in the NE sampling region, as shown in Figure 2. Note that all locations exhibited average porewater concentrations ∑PAH < 1 µg/L, and 50% of more of the porewater concentrations were often associated with DBF and 2-MNP in 2010. Three-ring and larger PAHs generally totaled less than 50 ng/L in 2010. The PAHs observed in the northwestern zone in 2010 were likely associated with sediment intermixed into the cap during placement in 2005 or as a result of equilibration with overlying water. No distinct profiles, such as higher concentrations at depth due to the higher concentrations in the contaminated sediment below the cap, were noted.

Samplers in the NW region exhibited porewater concentrations in 2018 that were generally similar or lower than those observed during the baseline 2010 sampling event. There was an average 45% decrease in ∑PAH in sediment porewater in the NW region with a 23% decrease in the higher-molecular-weight PAHs (three rings or greater).

Porewater concentrations in the NE section, however, were generally higher in 2018 than in 2010. This is shown by the different colored locations in Figure 2. Eleven out of the 12 individual sampling locations showed increased porewater concentrations between 2010 and 2018, with the highest increases near the shore and in the eastern and central portions of the NE area (notably locations 17, 18, 20, 21, 22, 24 which all showed increases in concentration more than a factor of 2 over 2010). In 2018, the three-ring and larger PAHs ranged from 134 to 408 ng/L at the NE locations of 16, 17, 18, 20, and 24, while all other locations in the NE area showed under 100 ng/L for three-ring and larger PAHs. Unlike 2010, the porewater concentrations were also highest at the bottom of the profiles, i.e., near the bottom of the cap layer and extending into the underlying contaminated sediment.

Table 1 shows the differences in ∑PAH between 2010 and 2018 at each location. Such a comparison does not reflect any variations in depth in the cap, nor does it recognize the typically higher mobility and higher porewater concentrations of low-molecular-weight compounds compared to the lower porewater concentrations and much lower mobility of the higher-molecular-weight PAHs. The comparison does provide a crude picture of the magnitude of changes between 2010 and 2018 and indicates that the bulk of the increased observed concentrations were in the NE region.

The conclusion from the 2018 observations were that although porewater PAH concentrations in the cap—and particularly near the cap–water interface—remained relatively low, higher concentrations were detected in the lower portions of the porewater sampler potentially as a result of migration into the bottom of the cap from below or as a result of the samplers being inserted into the underlying contaminated sediments. Table 2 summarizes the measured porewater concentrations in 2018, including ∑PAH averaged over depth and the concentrations for three selected PAHs in the top 10 cm and bottom 10 cm at each location. The individually identified PAHs in Table 2, phenanthrene (LogK_ow_ = 4.74), fluoranthene (LogK_ow_ = 5.29), and chrysene (LogK_ow_ = 5.9) represent compounds detected at most locations and cover a wide range of hydrophobicity which is related to sorption and contaminant migration rates.

### 3.2. Estimating Groundwater Upwelling Velocity from Performance Reference Compounds

The higher concentrations at the bottom of the porewater concentration profiles in 2018 suggested that an evaluation of future migration would assist in evaluating the long-term performance of the cap layer. PRCs were employed to estimate the degree of equilibration of target compounds using the methods of Shen and Reible [15]. As indicated in Lampert et al. [16], however, PRC release from thin PDMS fibers is normally controlled by external mass-transfer resistances. Thus, the PRC release can also be used to estimate the magnitude of those external mass-transfer resistances and—indirectly—the groundwater upwelling rates.

Kimura [9] examined the advective/diffusive transport of contaminants from a cylindrical source in a porous media subjected to a uniform velocity profile. With z being the vertical direction (along the axis of the cylindrical PDMS fiber holder, with z=0 being at the bottom of the PDMS fiber holder), the theory estimates the effective mass-transfer coefficient at the surface of the PDMS fiber ($k\;\left[\mathrm{cm}/d\right]$) subject to the velocity along the fiber ($U_{z}$) and the diffusion ($D_{eff}$) in the surrounding porous medium. In this analysis, the dimensionless Peclet number (ratio of advective to diffusive transport) and Sherwood number (dimensionless mass flux) are defined as

(3) $$Pe_{z}=\frac{U_{z}z}{D_{eff}}\;Sh=\frac{kz}{D_{eff}}=\frac{Flux}{C_{0}-C_{\infty }}\frac{z}{D_{eff}}$$

The resulting model (Appendix A) suggests that the mass-transfer coefficient is dependent upon the ratio of the vertical distance to the local curvature of the fiber (or in this case the fiber holder). The key parameter is ξ which is related to the chemical concentration boundary layer thickness relative to the radius of the cylinder holding the PDMS fiber.

(4) $$\xi =\frac{z}{r_{0}R_{f}\sqrt{Pe}}$$

Here, $r_{0}$ is the radius of the fiber holder and $R_{f}=\varepsilon +\rho_{b}K_{d}$ is the ratio of the total concentration in the medium r to that in the porewater (or retardation factor), where ε is the void fraction *t*, $\rho_{b}$ is the bulk density of the cap media, and $K_{d}$ is the partition coefficient between the cap media and porewater (estimated by $K_{oc}f_{oc}$, the product of the organic carbon based partition coefficient and the fraction organic carbon in the cap media). The thickness of the concentration boundary layer is reduced by sediment sorption effects and leads to the boundary layer being effectively “flat” on the surface of the PDMS holder, that is, ξ≪1, and the limit of forced convection on a flat surface in a porous medium applies to the release of the PRCs from the fiber (or the uptake of the target compounds). Using typical values of all parameters the value of ξ is less than 0.1 over the entire 1 m sampler length for the PAHs of interest (see Appendix A). Thus, under locally flat conditions, the mass transfer from the fiber to the porous medium is described by [18]. See also Supplementary Materials.

(5) $$Sh=\frac{1}{\sqrt{\pi }}Pe_{z}^{\frac{1}{2}}$$

The local mass-transfer coefficient, k, in Sh can be obtained from the release of PRCs from the passive sampler upon retrieval (Equation (6)).

(6) $$V\frac{dC_{PRC,fiber}}{dt}=kS\left(C_{PRC}{|}_{\infty }-C_{PRC}{|}_{0}\right)$$

where V is the volume of the SPME fiber layer [${\mathrm{cm}}^{3}$], S is the surface area [${\mathrm{cm}}^{2}$] of the PDMS, and the gradient of concentration is the difference between the total concentration in the surrounding medium (i.e., mass of PRC in the solid and the liquid phases) and the media at the surface of the PDMS. Note that $C_{PRC}{|}_{\infty }=0$ since the PRCs are not naturally present.

To obtain the total concentration in the porous media ($C_{PRC}$), consider that the PRC released from the polymer equilibrates with the adjacent sediment, where the total concentration includes both a dissolved and sorbed component (Equation (7)).

(7) $$C_{PRC}=\varepsilon \frac{C_{PRC,fiber}}{K_{fiber}}+\rho_{b}K_{d}\frac{C_{PRC,fiber}}{K_{fiber}}$$

where ε is the porosity and $\rho_{b}$ is the bulk density [kg/L] of the capping material, $K_{fiber}$ is the fiber-water partitioning coefficient [14], and $K_{d}\;\left[L/\mathrm{kg}\right]$ is the sediment–water partitioning coefficient derived from Thomas et al. [17]. Here, we estimate $K_{d}$ using the linear $K_{oc}f_{oc}$ relationship in the cap material in which the PDMS is inserted. From Equation (5) above, integrating assuming a constant mass-transfer coefficient, $k\;\left[\mathrm{cm}/d\right]$, yields:

(8) $$k=-\ln\left(\frac{C_{PRC,t}}{C_{PRC,0}}\right)\frac{V}{St}\frac{K_{fiber}}{\varepsilon +\rho_{b}K_{d}}=-\ln\left(1-f_{ss}\right)\frac{V}{St}\frac{K_{fiber}}{\varepsilon +\rho_{b}K_{oc}f_{oc}}$$

where $C_{PRC,t}$ is the PRC mass remaining on the polymer layer after an exposure time, $t\;\left[\mathrm{days}\right]$; and $C_{PRC,0}$ is the initial concentration of PRC concentration. At steady state, $C_{PRC,\infty }=0$, but none of the PRCs achieved complete equilibration with the surrounding media due to the relatively short (14-day) exposure time. The fractional approach to steady state, $f_{ss}$, varied between approximately 0.75 for the lightest PRC, d10-fluoranthene; to as low as 0.2 for the heaviest, d14-dibenzo[a,h]anthracene. For the purposes of the estimation of a mass-transfer coefficient and upwelling velocity, it is important that the PRCs do not approach equilibrium, at which point velocity estimation is no longer possible.

**Figure 4:**
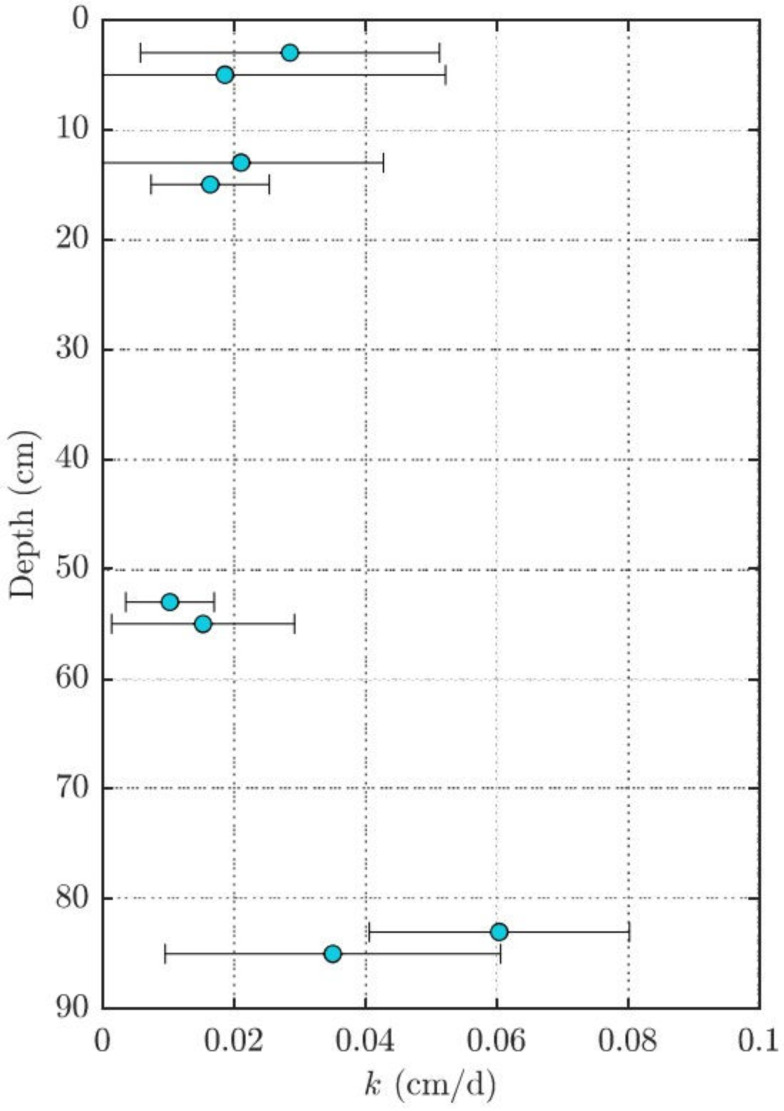
Local mass-transfer coefficient (k, cm/d) at depth of d-PAH performance reference compounds at sample location 20 (S20) within the NE sampling locations. The standard deviation in mass transfer reflects the uncertainty in PRC quantified from the PDMS passive samplers.

The local mass-transfer coefficient used to calculate the upwelling velocity was obtained from the PRC losses at each point of the sampler and at each sampling location. Figure 4 shows the calculated mass-transfer coefficient, k, for a representative location, 20, in the NE portion of the site. Location 20 was chosen because it is representative of the locations that exhibit both elevated concentrations in the bottom of the profile but also upwelling velocities typical of the NE area. The relative uncertainty in mass-transfer coefficient in Figure 4 reflects the uncertainty in PRC quantified from the PDMS passive samplers.

By solving for the advective transport, $U_{z}$, from Pe (Equation (3)) and relating Sh to the localized mass-transfer coefficient, the upwelling velocity can be determined (Equation (9) see Appendix A).

(9) $$U_{z}=k^{2}\pi \left(\frac{z}{D_{eff}}\right)$$

**Figure 5:**
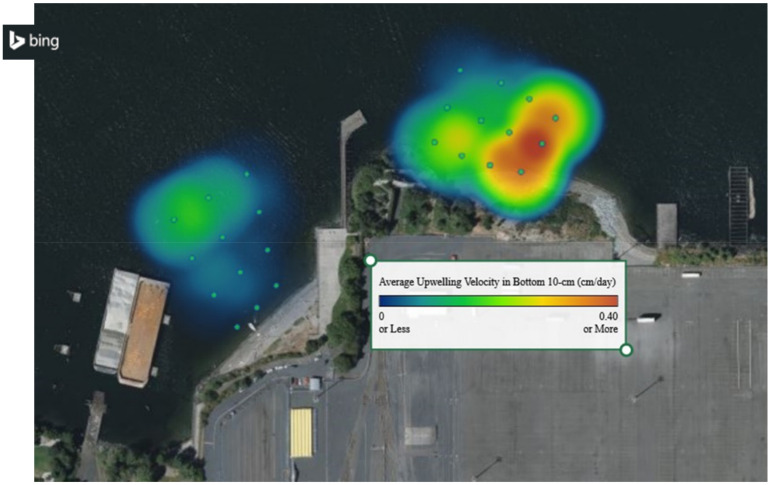
A heat map of the estimated distribution of upwelling groundwater flow (cm/day) averaged across the bottom 10 cm of the SPME sampler within the NW and NE sampling locations. The dots indicate the 24 SPME sampling stations. Microsoft product screen shot(s) reprinted with permission from Microsoft Corporation.

The upwelling velocity from the contaminated sediment beneath the cap was taken to be defined by that estimated at the bottom of the sampler that was likely not significantly influenced by near-surface tidal and wave fluctuations. The average groundwater velocity in the NW portion of the site was estimated to be about 0.09 cm/day (32 cm/year) while the average in the NE portion of the site was about 0.03 cm/day (13 cm/year). The top 10 cm in both areas exhibited an average of 4–5 cm/day, reflecting the greater movement due to tidal and wave action. Figure 5 shows a heat map of the estimated upwelling groundwater flow determined from Equation (9) averaged across the bottom 10 cm of the SPME passive sampler.

**Figure 6:**
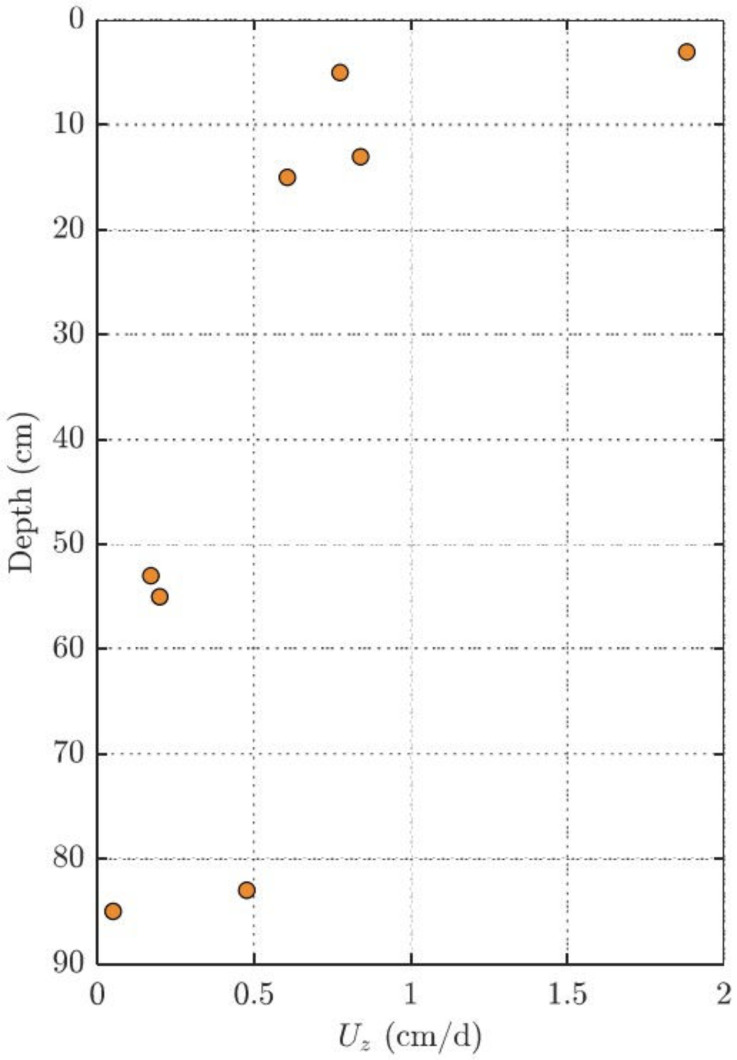
Upwelling velocity ($U_{z}$, cm/d) in the vertical direction at sample location 20 (S20) within the NE sampling section.

The upwelling velocity is the highest along the shoreline where hydraulic gradients are expected to be the greatest, and in those areas where the greatest increases in porewater concentration were observed. This provides support for the use of the model to estimate upwelling velocities. The upwelling velocity along the length of the sampler at location 20 is shown in Figure 6.

### 3.3. Modelled PAH Migration in the Cap

The observed porewater concentrations at the bottom of the profiles in the NE portion of the site in 2018 combined with groundwater upwelling suggest that there is likely to be contaminant migration over time, and the one-dimensional fate and transport software, CapSim [10], was used to estimate that migration. The modeling was focused on location 20 as a representative location showing both elevated concentrations at the bottom of the profile and substantial groundwater upwelling. Key CapSim parameters used in the simulation are included in Table 3.

**Figure 7:**
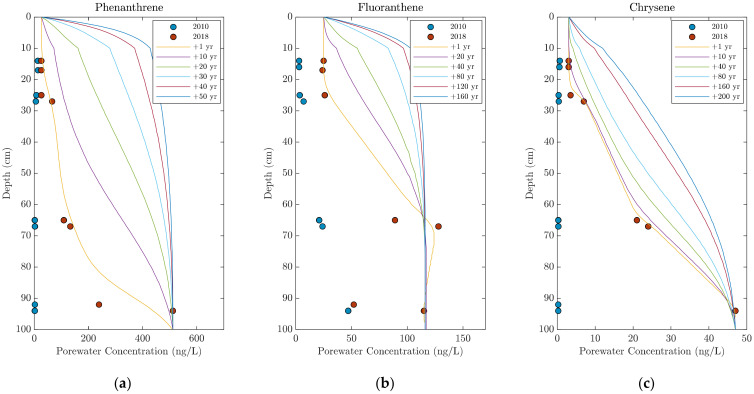
Depth-profile porewater concentrations from 2010 (blue circles) and 2018 (red circles) of (**a**) phenanthrene, (**b**) fluoranthene, and (**c**) chrysene at sampling location #20. Each graph has modeled contaminant transport through the cap based on the calculated mass-transfer coefficient, k, and upwelling velocity, $U_{z}$, of the d-PAH performance reference compounds.

Figure 7 shows the porewater depth profiles for phenanthrene, fluoranthene, and chrysene at individual depth intervals and modeled depth profiles based on the upwelling velocity, $U_{z}$, calculated over the bottom half of the sampler as shown in Figure 6 (0.21 cm/day), and the initial porewater concentration defined by 2018 measurements.

Based on the modeled results, phenanthrene (Figure 7, Phenanthrene) is expected to migrate relatively rapidly through the cap, and porewater concentrations over most of the cap should be similar to the concentrations at the bottom within 50 years if no degradation is assumed. Phenanthrene is a PAH that is subject to biological degradation under aerobic conditions, however, and this will likely lead to significant reductions in phenanthrene concentrations in the near-surface sediments. Significantly less migration of the more hydrophobic and more sorbing fluoranthene and chrysene is expected with porewater concentrations of fluoranthene becoming approximately uniform—except for the surface layer—within 200 years, while an even longer period would be expected to be required for chrysene and other high-molecular-weight PAHs. Note that these simulations assume that there is no degradation and that the source concentrations of PAHs at the bottom of the cap is not depleted over time.

## 4. Discussion

Passive samplers were deployed at the Pacific Sound Resources (PSR) Superfund Site (the Site) in Puget Sound, West Seattle, WA to measure near-surface contaminant porewater concentrations using solid-phase microextraction (SPME) by sorption onto polydimethylsiloxane (PDMS). PRCs of d-PAH compounds were used to assess the degree of equilibration of the target compounds. The objective of the study was to evaluate whether contaminated interstitial and groundwaters could be negatively impacting surficial sediment cap or surface water quality. The passive samplers were deployed in areas where potentially contaminated groundwater may discharge to surface waters through the sediment cap. By measuring profiles of porewater concentration, the contaminant in the mobile phase (water) can be directly assessed and migration from underlying source areas into the bottom layers of a cap can be directly measured without waiting for breakthrough of contamination at the surface.

The release of the d-PAH PRCs also provided a means of estimating upwelling velocities in the cap. The local Sherwood number can be estimated via passive sampling and used to estimate effective Peclet number or velocity, and this was used to predict long-term migration of contaminants of concern in the cap layer. One important assumption of the method to estimate groundwater upwelling velocity is that the cylindrical holder is locally “flat”. This is valid as a result of the sorption onto the surrounding sediment/cap material which keeps the PRC concentration boundary layer around the sampler “thin” compared to the diameter of the sampler. The method developed provides an estimate of groundwater upwelling velocity; however, there are no independent estimates to evaluate the validity of the results. The method does, however, suggest that the maximum upwelling occurs in the locations where the highest porewater concentrations were measured in 2018 relative to 2010. These locations also tended to be located in the shallowest water near the shore, where the effects of groundwater gradients and tidal variations in water depth and hydraulic head are expected to be the greatest. The correspondence between the locations with the greatest upwelling velocities and the largest increases in porewater concentrations and the greatest expected influence of hydraulic gradients suggests that the method at least provides an indication of the relative transport or mixing rates at the various locations.

The method also cannot indicate flow direction; that is, it does not differentiate between upwelling or downwelling. The observed groundwater gradients are generally from the upland area to Puget Sound, however, so the net movement is expected to be toward the Sound. The tidal fluctuations up and down in the near-surface sediment/cap, however, likely suggest that the observed estimated velocity in the near-surface environment represents a magnitude that includes water being both retained and lost from Puget Sound.

The estimated groundwater migration rates were used to predict future cap performance using CapSim. A key parameter for the future performance predictions, in addition to the groundwater migration rates, is the partition coefficient in the sediment and/or capping material, which can be directly measured through samples collected via cores or estimated by compound properties and the fraction organic carbon in the capping layer.

## 5. Conclusions

This work shows the ability to determine the groundwater upwelling velocity and contaminant flux using the rate of equilibration of performance refence compounds. The approach to estimation of the groundwater upwelling velocity is a novel use of the PRCs that can be employed at other locations.

The work also illustrates the capability of using measured porewater concentration profiles to not only indicate current cap performance but also to provide a basis for prediction in the future. In this case, the estimated upwelling velocities allowed estimation of the migration of currently observed porewater concentrations through the cap over time using CapSim. The time predictions are improved by profile monitoring over long time periods so that contaminant migration into the cap layer from source areas below can be assessed, as well as ultimately provide data that could be used to evaluate or calibrate models. In this case, the lack of significant porewater concentrations in the cap in 2010 means that only the 2018 data could be used to predict long-term performance. Subsequent sampling, however, could continue to be used to update and refine the model predictions.

## Figures and Tables

**Table 1:** Sum of porewater PAHs averaged over depth in 2010 and 2018.

Sample Location		∑PAH (2010) ng/L	∑PAH (2018) ng/L
Northwest	1	70	121
	2	170	92
	3	34	125
	4	97	85
	5	490	124
	6	71	27
	7	104	21
	8	170	-
	9	89	-
	10	66	66
	11	315	111
	12	67	12
Northeast	13	89	384
	14	66	186
	15	75	324
	16	58	809
	17	74	690
	18	68	567
	19	170	33
	20	83	876
	21	99	413
	22	47	439
	23	56	378
	24	86	576

**Table 2:** Summary of ∑_16_PAH averaged over depth and the concentrations for three selected PAHs in the top 10 cm and bottom 10 cm at each location sampled in 2018. Estimated velocity at each location is also included (discussed later).

		Top 10-cm Depth (0–10 cm)				Bottom 10-cm Depth (80–90 cm)			
Station ID	∑PAH_16_ ng/L	U_z_ (cm/d)	Porewater Concentration (ng/L)			U_z_ (cm/d)	Porewater Concentration (ng/L)		
			Phenanthrene	Fluoranthene	Chrysene		Phenanthrene	Fluoranthene	Chrysene
Northwest									
1	121	1.4	2.5	<1.0	<0.1	0.02	11.6	9.8	0.8
2	92	2.4	5.7	21.6	2.3	0.08	1.3	7.8	2.5
3	125	8.3	<1.0	10.1	<0.1	0.10	0.1	<1.0	<0.1
4	85	1.7	15.7	20.3	<0.1	0.01	12.3	8.1	<0.1
5	124	5.7	0.8	9.9	1.1	0.02	48.6	12.4	1.2
6	27	10.1	2.1	7.9	<0.1	0.02	12.1	9.1	<0.1
7	21	6.5	1.3	14.1	2.5	0.00	<1.0	<1.0	<0.1
10	66	3.4	15.7	20.5	<0.1	0.02	<1.0	8.1	<0.1
11	111	2.7	1.2	0.1	<0.1	0.02	61.2	12.4	1.2
12	12	10.4	1.3	14.1	1.8	0.01	<1.0	<1.0	0.8
Northeast									
13	384	6.7	15.5	9.3	<0.1	0.02	8.3	16.5	1.1
14	186	<0.1	<1.0	<1.0	<0.1	0.10	10.9	<1.0	<0.1
15	324	4.6	8.6	6.4	<0.1	0.12	1.0	<1.0	<0.1
16	809	2.8	28.3	49.6	1.7	0.01	44.9	38.2	1.0
17	690	6.6	147.7	162.5	2.1	0.08	28.8	13.9	1.6
18	567	1.1	18.3	32.9	0.7	0.02	461.4	132.2	<0.1
19	33	2.2	2.2	18.7	<0.1	0.06	6.6	27.3	<0.1
20	876	1.3	26.3	24.6	3.1	0.26	350.5	78.3	46.8
21	412	2.7	39.6	39.3	<0.1	0.05	24.6	12.3	<0.1
22	439	0.7	21.9	25.5	0.9	0.02	38.3	17.2	0.8
23	378	1.0	50.8	21.2	<0.1	0.03	17.2	11.6	<0.1
24	576	14.9	26.2	54.6	1.7	0.32	84.1	159.3	14.4

**Table 3:** Key model parameters for simulations shown in Figure 7.

Layer	Depth (cm)	*f_oc_*	U_z_ (cm/day)	Boundary Condition	Comments
Sediment	10	0.01	0.21	Mass transfer	Bioturbation in top 5 cm at 2 cm^2^/year
Sand/Gravel	90	0.003	0.21	Constant Concentration	No depletion in bottom concentration
**Contaminant**	***K_oc_***		**Initial Concentrations**		
Phenanthrene	3.93		2018 porewater concentrations as shown in Figure 7—Local equilibrium with adjacent solids assumed		
Fluoranthene	4.51				
Chrysene	5.09				

## Data Availability

The CapSim model and its inputs and outputs are available from the corresponding author.

## Supplementary Materials

The following supporting information can be downloaded at: https://www.mdpi.com/article/10.3390/toxics10030106/s1, Table S1: Priority Pollutant Polycyclic Aromatic Hydrocarbons (PAHs) + 2-methylnaphthalene (2MNP) and dibenzofuran (DBF) Retention times Using High Performance Liquid Chromatography (HPLC) with fluorescence detection (FLD). Upwelling velocity derived from Peclet-Sherwood Relations. References [22,23,24] are citied in the Supplementat Materials.

## Appendix A. Estimating Groundwater Upwelling Velocity from Performance Reference Compounds

Sediment characteristics of Puget Sound are defined below.

$$\varepsilon =0.5\;\;\rho_{b}=\left(1-\varepsilon \right)*2.6\frac{g}{{\mathrm{cm}}^{3}}\;\;f_{oc}=0.003\;\;Rf=\varepsilon +\rho_{b}K_{d}$$

where ε represents the sediment porosity, $\rho_{b}$ is the sediment bulk density, $f_{oc}$ is the fraction of organic carbon, and Rf is the retardation factor.

The following transport and SPME parameters were also utilized to describe the effective diffusion coefficient for the surrounding porous media (D, which is the diffusion coefficient in water multiplied by $\varepsilon^{4/3}$ to account for porosity and tortuosity [19,20]), upwelling velocity (U), the radius of the SPME fiber holder ($r_{o}$) and the organic carbon partitioning coefficient ($K_{oc}$) for phenanthrene, fluoranthene, and chrysene, respectively, adapted from Baker [21].

$$D=5*10^{-6}\frac{\mathrm{cm}}{s}*0.5^{\frac{4}{3}};\;U=1\frac{\mathrm{cm}}{d};\;r_{0}=\frac{0.375}{2}in=0.476\;\mathrm{cm};\;K_{oc}=\left\{\begin{array}{c} 10^{4.37}\\ 10^{4.87}\\ 10^{5.42} \end{array}\right.\frac{L}{\mathrm{kg}}$$

The dimensionless Peclet number (ratio of advective to diffusive transport) and Sherwood number (dimensionless mass flux) are defined as

$$Pe_{z}=\frac{U_{z}z}{D_{eff}}\;Sh=\frac{kz}{D_{eff}}=\frac{Flux}{C_{0}-C_{\infty }}\frac{z}{D_{eff}}$$

where z is along the axis of the cylinder in the vertical direction, in this case towards the sediment–water interface.

The mass-transfer coefficient is dependent upon the ratio of the vertical distance to the local curvature of the fiber holder. The applicable parameter is ξ, defined by

$$\xi =\frac{z}{r_{o}R_{f}\sqrt{Pe}}$$

This differs from the definition given by [9] in that includes $R_{f}=\varepsilon +\rho_{b}K_{d}$ associated with sorption in the surrounding media. An examination of transient diffusion in the radial direction shows that the radial extent of concentration migration decreases in inverse proportion to $R_{f}$. This results in a thin concentration “boundary” layer next to the cylindrical fiber which supports the locally “flat” cylinder assumption.

Using typical values of all parameters described above (with upwelling velocity of the order of ~1 cm/d), the value of ξ is less than 0.1 over the entire 1 m sampler length for the PAHs of interest (phenanthrene, fluoranthene, and chrysene, respectively).

$$R_{f}=\left\{\begin{array}{c} 92\\ 290\\ 1026 \end{array}\right.\;\;Pe=583\;\xi =\left\{\begin{array}{c} 0.095\\ 0.03\\ 0.008 \end{array}\right.$$

Since ξ≪1, locally flat conditions can be used and the mass transfer from the fiber to the porous medium is defined by

$$Sh=\frac{1}{\sqrt{\pi }}Pe_{z}^{1/2}$$

The local mass-transfer coefficient can then be obtained from the release of PRCs from the passive sampler upon retrieval.

The mass-transfer coefficient, k, can be obtained from the fraction of performance reference compounds, $f_{PRC}$, lost from the passive-sampling fiber layer.

$$F_{PRC}=V\frac{dC_{PRC}}{dt}=kS\left(\bar{C_{PRC}}{│}_{z\to \;\infty }-\bar{C_{PRC}}{│}_{z=0}\right)$$

where V is the volume of PDMS fiber, S is the surface area of PDMS fiber, $C_{PRC}$ is the concentration in fiber, $\bar{C_{PRC}}$ is the overall concentration in sediment (i.e., mass of PRC per total volume of sediment), and $K_{PDMS}$ is the water–PDMS partition coefficient.

At z=0, the PRC release from the polymer equilibrates with the adjacent sediment of porosity ε and bulk density $\rho_{b}$ and the concentration includes both a term for porewater and for sediment partition coefficient $K_{d}$.

$$\bar{C_{PRC}}=\frac{\varepsilon +\rho_{b}K_{d}}{K_{PDMS}}C_{PRC}$$

Solving for the mass-transfer coefficient from the fraction of PRC, we obtain:

$$k=-\ln\left(\frac{C_{PRC}\left(t\right)}{C_{PRC}\left(t=0\right)}\right)\frac{V}{St}\frac{K_{PDMS}}{\varepsilon +\rho_{b}K_{d}}=-\ln\left(1-f_{ss}\right)\frac{V}{St}\frac{K_{fiber}}{\varepsilon +\rho_{b}K_{oc}f_{oc}}$$

By solving for the advective transport, $U_{z}$ from the Peclet number (Pe) and relating the Sherwood number (Sh) to the localized mass-transfer coefficient, the upwelling velocity can be determined from the use of PRCs in SPME passive samplers.

$$U_{z}=k^{2}\pi \left(\frac{z}{D_{eff}}\right)$$
