# Supplementary material for: In Situ Passive Sampling to Monitor Long Term Cap Effectiveness at a Tidally Influenced Shoreline
Source: Toxics. 2022 Feb 23;10(3):106. doi: 10.3390/toxics10030106 (PMC8948705; doi:10.3390/toxics10030106)
Supplement: Supplementary file 1 [file toxics-10-00106-s001.zip › toxics-1558903-supplementary.pdf]

toxics

MDPI

Supplementary Materials: Title

Retention Times for Target Compounds

Table S1. Priority Pollutant Polycyclic Aromatic Hydrocarbons (PAHs) + 2-methylnaphthalene (2MNP) and dibenzofuran (DBF) Retention Times using High Performance Liquid Chromatography (HPLC, Agilent 1260) with fluorescence detection (FLD).

Priority Pollutant PAHs (PAH_{16}) + 2MNP +DBF	Retention Time (minutes)
Naphthalene	7.789
2-Methylnaphthalene	9.821
Fluorene	10.743
Dibenzofuran	11.021
Acenaphthene	11.127
Phenanthrene	11.692
Anthracene	12.450
Fluoranthene	14.543
Pyrene	15.674
Chrysene	19.254
Benz[a]anthracene	19.949
Benzo[b]fluoranthene	25.658
Benzo[k]fluoranthene	26.616
Benzo[a]pyrene	27.732
Dibenzo[a,h]anthracene	33.892
Benzo[g,h,i]perylene+Ideno[1,2,3-cd]pyrene	36.795

Note: acenaphthylene is not detectable by FLD and benzo[g,h,i]perylene and indeno[1,2,3-cd]pyrene co-elute.

Upwelling velocity derived from Peclet – Sherwood Relations

1.0. Diffusive – Advective Transport in Porous Media

If we consider advective and diffusional transport in a porous media adjacent to a cylinder that is assumed locally "flat" (see section 4) where transport in the direction perpendicular to the fiber (x) is assumed to occur only by diffusion and advection is assumed to dominate the transport along the fiber (z), the governing equation can be written,

$$U_z \frac{dC}{dz} = D_{eff} \frac{d^2C}{dx^2}$$

We can non-dimensionalize concentration (C) by

$$\theta = \frac{C_* - C_0}{C_\infty - C_0}$$

2.0. Peclet – Sherwood Relationship

The relative magnitude of advective to diffusive mass transfer processes is expressed by the Peclet number (Pe).

$$Pe_z = \frac{U_z z}{D_{eff}}$$

Introducing a similarity variable suggested by the scaling law:

$$\eta = \frac{x}{z} Pe_z^{1/2}$$

Then the equation becomes;

$$\theta'' + \frac{1}{2}\eta\theta' = 0$$

With boundary conditions

$$\theta(0) = 0$$

$$\theta(\infty) = 1$$

Incorporating the initial conditions to the previous equation, separation of variables can be used to generate the following expression.

$$\left(\frac{d\theta}{d\eta}\right)_{\eta=0} = \pi^{-\frac{1}{2}} = 0.564$$

The local Sherwood number (Sh), which represents the ratio of the convective mass transfer to the rate of diffusive mass transport can be estimated by the Pe.

$$Sh = \frac{kz}{D_{eff}} = \frac{Flux}{C_0 - C_\infty}\frac{z}{D_{eff}} = \left(\frac{d\theta}{d\eta}\right)_{\eta=0} Pe_z^{1/2} = 0.564Pe_z^{1/2}$$

This is the well-known solution for advective-diffusive transport in a porous media adjacent to a flat surface (Bejan 2013). This is also the solution for the case for advective-diffusive transport along a cylinder in porous media in the limit as the cylinder can be treated as "locally flat", that is, that the concentration profile diffusing away from the cylinder is not influenced by the curvature of the cylinder (Kimura 1988).

3.0. Determining the mass transfer coefficient, $\boldsymbol{k}$, and upwelling velocity, $\boldsymbol{U_z}$

The mass transfer coefficient, k, can be obtained from the fraction of performance reference compounds, f_{PRC}, lost from the passive sampling fiber layer.

$$f_{PRC} = V\frac{dC_{PRC}}{dt} = kA\left(\overline{C_{PRC}}\,|_{z\to\infty} - \overline{C_{PRC}}\,|_{z=0}\right)$$

Where V is the volume of PDMS fiber, A is the surface area of PDMS fiber, C_{PRC} is the concentration in fiber, and $\overline{C_{PRC}}$ is the total concentration in sediment, K_{PDMS} is the water-PDMS partition coefficient.

At $z = 0$ the PRC release from the polymer equilibrates with the adjacent sediment of porosity ε and bulk density ρ_b and the concentration includes both a term for porewater and for sediment partition coefficient K_d.

$$\overline{C_{PRC}} = \frac{\varepsilon + \rho_b K_d}{K_{PDMS}} C_{PRC}$$

Solving for the mass transfer coefficient from the fraction of PRC we get;

$$k = -\ln\left(\frac{C_{PRC}(t)}{C_{PRC}(t=0)}\right)\frac{V}{At}\frac{K_{PDMS}}{\varepsilon + \rho_b K_d}$$

Based on the definition of the Peclet number, Sherwood number, and their relationship. The groundwater upwelling velocity (U_z) can be expressed as a function of effective diffusivity:

$$U_z = \frac{z}{D_{eff}}\left(\frac{k}{0.564}\right)^2$$

4.0. Assumption of Locally "Flat" Cylindrical Fiber

The effect of the cylindrical character of the fiber can be evaluated by considering transient diffusion away from a cylinder in an infinite medium. This process is governed by the equation

$$(\varepsilon + \rho_b K_d)\frac{\partial C}{\partial t} = R_f \frac{\partial C}{\partial t} = \frac{\mathfrak{D}}{r}\frac{\partial}{\partial r} r \frac{\partial C}{\partial r}$$

Nondimensionalizing

$$\frac{\partial C}{\partial \tau} = \frac{1}{r^*}\frac{\partial}{\partial r^*} r^* \frac{\partial C}{\partial r^*}$$

Where $\tau = \frac{\mathfrak{D}t}{r_0^2}$ and $r^* = \frac{rR_f}{r_0}$. The latter non-dimensionalization emphasizes that the radial influence of the diffusion is very small due to the sorption onto the surrounding media. That is, for a given value of r^* , the physical distance $r = \frac{r^* r_0}{R_f}$ which is much smaller than simply $r^* r_0$ and the assumption of locally flat cylinder is asymptotically valid for strongly sorbing contaminants such as $R_f \to \infty$.

A solution to the non-dimensional equation given by Crank (Crank 1975) provides the following solution for the flux, valid at short times

$$Flux = \frac{D_{eff}(C_0 - C_\infty)R_f}{r_0}\left(\pi \frac{D_{eff} t R_f^{\,2}}{r_0^{\,2}}\right)^{-1/2} = \frac{D_{eff}(C_0 - C_\infty)R_f}{r_0}\frac{r_0}{\pi^{1/2} D_{eff}^{\;1/2} R_f}$$

$$Flux = \frac{D_{eff}^{\;1/2}(C_0 - C_\infty)}{\pi^{1/2}}$$

Recognizing that time available for diffusion radially in a fluid parcel moving up the cylinder is given by z / U_z , the Sherwood number can be written

$$Sh = \frac{kz}{D_{eff}} = \frac{Flux}{C_0 - C_\infty}\frac{z}{D_{eff}} = \frac{D_{eff}^{1/2}(C_0 - C_\infty)U_z^{1/2}}{(C_0 - C_\infty)D_{eff}\pi^{1/2} z^{1/2}}$$

$$Sh = \frac{U_z^{1/2} z^{1/2}}{D_{eff}^{1/2}\pi^{1/2}} = \frac{1}{\pi^{1/2}} Pe_z^{\;1/2}$$

As before and the previous relationship between the upwelling velocity in the porous media around the passive sampling cylinder and the effective mass transfer coefficient holds.

References

1. Bejan, A. (2013). Convection heat transfer, John wiley & sons.
2. Crank, J. (1975). The mathematics of diffusion. Oxford, Clarendon Press.
3. Kimura, S. (1988). "Forced convection heat transfer about a cylinder placed in porous media with longitudinal flows." International journal of heat and fluid flow 9(1): 83-86.
